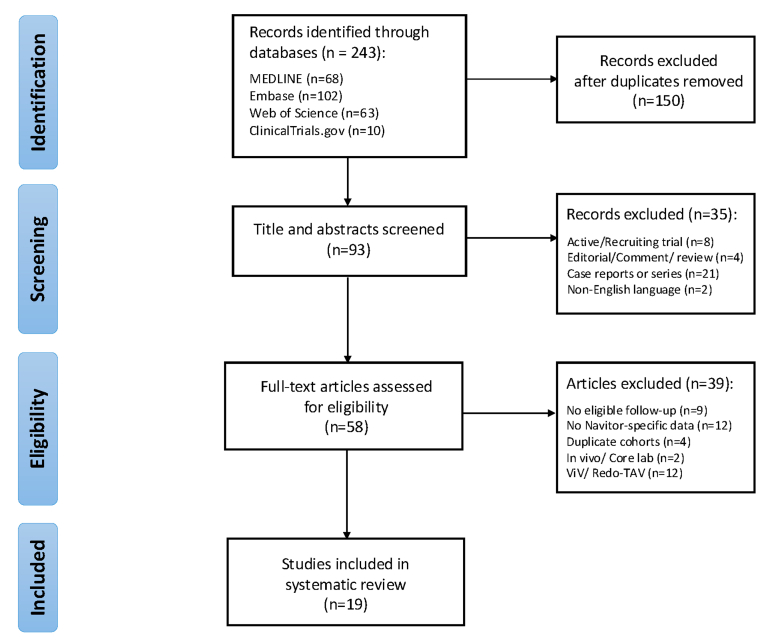# Midterm Outcomes of the Self-Expanding Navitor Transcatheter Heart Valve: A Systematic Review and Meta-Analysis

**DOI:** 10.1016/j.shj.2025.100750

**Published:** 2025-11-04

**Authors:** Sahar Samimi, Chloe Kharsa, Mangesh Kritya, Joe Aoun, Syed Zaid, Nadeen N. Faza, Stephen H. Little, Neal S. Kleiman, Michael J. Reardon, Sachin S. Goel

**Affiliations:** a Department of Cardiology, Baylor College of Medicine, Houston, Texas, USA; b Department of Cardiology, Houston Methodist DeBakey Heart and Vascular Center, Houston, Texas, USA; c Department of Cardiovascular Surgery, Houston Methodist Hospital, Houston, Texas, USA

**Keywords:** Meta-analysis, Navitor, TAVI, TAVR, Transcatheter aortic valve replacement, Transcatheter aortic valve implantation

## Abstract

• A 19-study meta-analysis (7743 ​patients) found high Navitor technical/device success.
• ≥Moderate PVL was low: 1.3% at 30 days and 0.6% at 1 year.
• Pacemaker implantation was 16.7% at 30 days and 20.4% at 1 year.
• Thirty-day outcomes were favorable: all-cause mortality 1.5% and stroke 1.7%.

The rapid evolution of transcatheter aortic valve replacement (TAVR) has catalyzed significant innovation in device design, particularly as indications extend to younger and lower-risk patients.^1^ With lifetime management emerging as a key priority, contemporary TAVR systems must optimize outcomes not only periprocedurally but also in the long term.

Among contemporary self-expanding transcatheter heart valves, the Navitor (Abbott Structural Heart) received U.S. Food and Drug Administration approval in 2023 for patients at high surgical risk and holds a European Conformité Européenne mark, extending its indication to all surgical risk categories.^2^ The device builds upon the design of its predecessor, the Portico valve, with the addition of an active outer fabric cuff (NaviSeal) intended to reduce paravalvular leak (PVL). Since its regulatory approvals, an increasing number of real-world data sets and multicenter registries have reported on the safety and performance of the Navitor valve. To synthesize these findings, we conducted a meta-analysis of investigations evaluating the 30-day and 1-year clinical outcomes of TAVR using Navitor in patients with severe aortic stenosis at high surgical risk.

This study-level meta-analysis was conducted in accordance with the Preferred Reporting Items for Systematic Reviews and Meta-Analyses guidelines (Supplemental Figure 1). A systematic search of PubMed, EMBASE, Web of Science, and ClinicalTrials.gov was performed through September 30, 2025, to identify studies reporting outcomes following TAVR with the Navitor valve, including both peer-reviewed articles and high-quality presentations from reputable cardiovascular scientific meetings (Supplemental Table 1).

Eligible studies enrolled adults undergoing TAVR for native aortic stenosis and reported 30-day and/or 1-year outcomes for the Navitor valve. We excluded non-English publications; case reports or case series with <10 patients; editorials, comments, and reviews; valve-in-valve and redo-TAVR cohorts; and studies without Navitor-specific outcomes. Permanent pacemaker implantation (PPI) was calculated only among patients without a prior pacemaker. When reports described overlapping populations, the most complete, nonduplicative data set was retained. Study quality was appraised using a modified Newcastle–Ottawa Scale for cohort studies (Supplemental Table 2). As this review used aggregated data from published studies, institutional ethics approval and patient consent were not required.

Meta-analyses were performed using the meta package in R (v4.3.2). Pooled event rates and 95% confidence intervals (CIs) were calculated using a random-effects model with inverse-variance weighting. For outcomes with an incidence of >5%, the logit transformation was applied, and for rarer outcomes, the Freeman-Tukey double arcsine. Heterogeneity was assessed using the I^2^ and τ^2^ statistics. Subgroup sensitivity analyses compared studies with bicuspid aortic valve (BAV) only and cohorts with tricuspid aortic valve, pooling 30-day PPI (logit) and ≥moderate PVL (Freeman–Tukey) using a random-effects inverse-variance model (restricted maximum likelihood, Hartung–Knapp) with outcome-specific denominators; differences between groups were tested by the Q test (p-interaction).

A total of 19 studies comprising 7743 patients were included.^1^, ^2^, ^3^, ^4^, ^5^, ^6^, ^7^, ^8^, ^9^, ^10^, ^11^, ^12^, ^13^, ^14^, ^15^, ^16^, ^17^, ^18^, ^19^ The pooled mean age was 81.8 years (95% CI: 80.6–83.1), 63.2% were female, and the pooled mean Society of Thoracic Surgeons score was 4.3% (95% CI: 2.7–6.7). At 30 days, technical success was 96.7% (95% CI: 95.2–97.8; k = 12; I^2^ = 0.9) and device success 91.1% (86.8–94.1; k = 9; I^2^ = 0.7); new PPI occurred in 16.7% (12.9–21.4; k = 17; I^2^ = 0.8). All remaining pooled 30-day event rates, including moderate or greater PVL (1.3% [0.5–2.3]; k ​= ​15; I^2^ ​= ​0.7), were ≤3% across studies (Figure 1, left panel). At 1 ​year, four studies reported PPI events, with a pooled rate of 20.4% (15.0–27.1; k = 4; I^2^ = 0.4). Among 5 studies reporting moderate or greater PVL events at 1 ​year, pooled moderate-or-greater PVL remained low at 0.6% (0.00–3.16; k = 5; I^2^ = 0.7). All-cause mortality occurred at 6.9% (0.9–17.5; k = 3; I^2^ = 0.9). All remaining pooled 1-year event rates remained low at ≤3% across studies (Figure 1, right panel).

**Figure 1:**
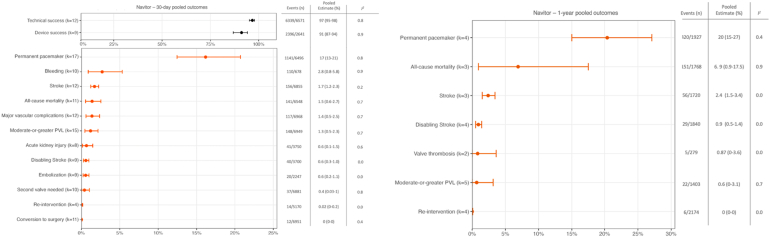
**Pooled 30-day and 1-year clinical outcomes following TAVR with the Navitor transcatheter heart valve.** Abbreviations: PVL, paravalvular leak; TAVR, transcatheter aortic valve replacement.

Two studies (n = 107) exclusively enrolled patients with BAV.^4^^,^^12^ In a 30-day sensitivity analysis comparing BAV with tricuspid aortic valve, PPI occurred in 17 (15.5%) in BAV vs. 1124 (16.8%) in tricuspid, and ≥moderate PVL in 2 patients (1.2%) in BAV vs. 146 (1.2%) in tricuspid. Despite the small number of BAV studies and events, there was no evidence of effect modification by valve morphology (p for interaction >0.05).

The Navitor system demonstrates strong early performance, with high technical and device success and consistently low rates of ≥moderate PVL at 30 days and 1 year, findings consistent with its active sealing cuff designed to mitigate PVL. In contrast, PPI remains frequent (17% at 30 days and 20% at 1 year), highlighting the need for further evaluation of strategies to mitigate conduction injury (e.g., implant depth optimization, cusp-overlap technique, and selective predilation/postdilation).

In the risk of bias assessment (modified Newcastle–Ottawa), the evidence consisted of nonrandomized, single-arm cohorts, which introduce confounding and at least moderate risks related to the representativeness of the studied population. Estimates should also be interpreted in light of variability in procedural protocols and the absence of patient-level data. One-year results were derived from few studies, k = 5 for ​≥moderate PVL and k = 4 for other outcomes, and often relied on outcome-specific denominators (particularly 1-year echocardiography), further warranting caution. Despite these limitations, this work represents the most comprehensive synthesis of Navitor clinical outcomes to date. Continued real-world surveillance and randomized head-to-head trials are needed to define long-term durability, reintervention risk, and strategies to reduce pacemaker rates.

## Ethics Statement

This systematic review and meta-analysis used only aggregated data from previously published studies; no new human or animal research was performed. Accordingly, ethics committee approval and informed consent were not required.

## Funding

The authors have no funding to report.

## Disclosure Statement

S.S. Goel reports a relationship with 10.13039/100000046Abbott that includes: consulting or advisory; and serves as a consultant for 10.13039/100004374Medtronic, JC Medical, 10.13039/100008497Boston Scientific, Abbott Structural Heart, and W. L. Gore & Associates. M.J. Reardon reports a relationship with 10.13039/100000046Abbott that includes: consulting or advisory. Other: M.J. Reardon has consultancy roles with 10.13039/100004374Medtronic, 10.13039/100008497Boston Scientific, 10.13039/100000046Abbott, and W. L. Gore & Associates. N.S. Kleiman is a local principal investigator for trials sponsored by 10.13039/100008497Boston Scientific, 10.13039/100004374Medtronic, 10.13039/100000046Abbott, and 10.13039/100006520Edwards Lifesciences. The other authors had no conflicts to declare.

## Figures and Tables

**Supplemental Figure 1:**