# Supplementary material for: Midterm Outcomes of the Self-Expanding Navitor Transcatheter Heart Valve: A Systematic Review and Meta-Analysis
Source: Struct Heart. 2025 Nov 4;10(1):100750. doi: 10.1016/j.shj.2025.100750 (PMC12741374; doi:10.1016/j.shj.2025.100750)

# Supplemental Table 1. Systematic Search Strategies for Navitor Valve Outcomes

| Database | Search Strategy | N |
| --- | --- | --- |
| PubMed | (("Navitor"[tiab] AND (valve[tiab] OR system[tiab])) AND (TAVR[tiab] OR TAVI[tiab] OR "transcatheter aortic val*"[tiab] OR valv*[tiab])) | 68 |
| Embase | (('navitor':ti,ab,kw AND (valve:ti,ab,kw OR system:ti,ab,kw)) AND (tavr:ti,ab,kw OR tavi:ti,ab,kw OR 'transcatheter aortic val*':ti,ab,kw OR valv*:ti,ab,kw)) | 102 |
| Web of Science | ALL=(Navitor AND (valve OR system OR platform) AND (TAVR OR TAVI OR "transcatheter aortic valve*" OR valv*)) | 63 |
| ClinicalTrials.gov | (Navitor AND (valve OR system) AND (TAVR OR TAVI OR transcatheter aortic)) | 10 |

# Supplemental Table 2. Modified Newcastle-Ottawa Quality Assessment Tool

| Author,  Year | Representativeness of the Population | Ascertainment of Exposure | Ascertainment of Outcome | Sufficient follow-up time | Adequacy of Follow Up | Overall Risk of Bias |
| --- | --- | --- | --- | --- | --- | --- |
| Worthley S.,  2025 | **** | **** | **** | **** | **** | 20/20 |
| Ruberti A.,  2025 | *** | **** | **** | **** | **** | 19/20 |
| Olivia O.,  2025 | * | **** | **** | *** | **** | 16/20 |
| Cammardella A., 2025 | ** | **** | **** | **** | **** | 18/20 |
| Campanella C.,  2025 | ** | **** | **** | *** | **** | 17/20 |
| Cannata S.,  2025 | *** | **** | **** | **** | **** | 19/20 |
| Garcia S.,  2025 | *** | **** | **** | *** | **** | 18/20 |
| Eckel CE,  2025 | *** | **** | **** | *** | **** | 18/20 |
| Casenghi M.,  2025 | *** | **** | **** | *** | **** | 18/20 |
| Iwata J.,  2025 | ** | **** | **** | *** | **** | 17/20 |
| Fujita K.,  2025 | ** | **** | **** | *** | **** | 17/20 |
| Alnasser S.,  2024 | *** | **** | **** | *** | **** | 18/20 |
| Ghoneem A.,  2024 | * | **** | **** | *** | **** | 16/20 |
| Bharucha AH,  2024 | **** | **** | **** | *** | **** | 19/20 |
| Shirai S.,  2024 | **** | **** | **** | *** | **** | 19/20 |
| Waksman R.,  2023 | **** | **** | **** | **** | **** | 20/20 |
| Reardon MJ,  2023 | **** | **** | **** | *** | **** | 20/20 |
| Eckel CE,  2023 | * | **** | **** | *** | **** | 16/20 |
| Corcione N.,  2022 | **** | **** | **** | *** | **** | 19/20 |

Supplemental Figure 1. PRISMA flow chart


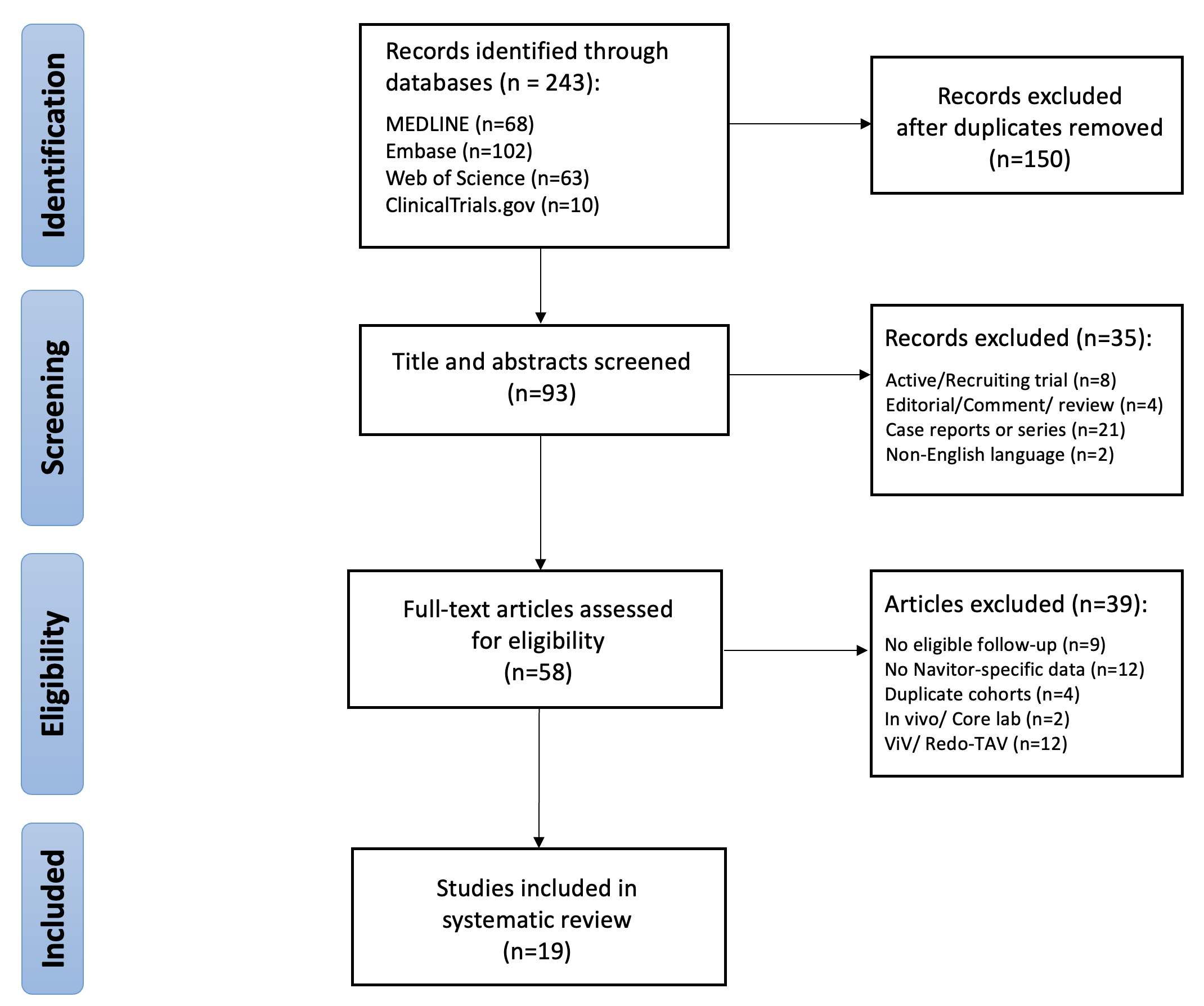

Supplement: Supplementary Material [file mmc1.docx]
